# Preliminary Assessment of Repellency and Toxicity of Essential Oils against *Sitophilus zeamais* Motschulsky (Coleoptera: Curculionidae) on Stored Organic Corn Grains

**DOI:** 10.3390/foods11182907

**Published:** 2022-09-19

**Authors:** Sawo Eesiah, Jianmei Yu, Beatrice Dingha, Barbara Amoah, Nona Mikiashvili

**Affiliations:** 1 Food and Nutritional Sciences Program, Department of Family and Consumer Sciences, North Carolina A&T State University, 1601 East Market Street, Greensboro, NC 27411, USA; 2 Department of Natural Resources and Environmental Design, North Carolina A&T State University, 1601 East Market Street, Greensboro, NC 27411, USA

**Keywords:** essential oil, insecticidal activity, organic corn grains, maize weevil

## Abstract

Insect infestation of cereal grains during post-harvest storage not only causes significant grain loss, but also reduces grain quality and makes grains more susceptible to mold infection. Synthetic pesticides are banned from being used in organic grain storage setting due to their high toxicity. The main insect damaging stored corn grains is maize weevil, *Sitophilus zeamais* Motschulsky (Coleoptera: Curculionidae). The purpose of this study was to evaluate insect repellency and insecticidal potentials of some generally recognized as safe (GRAS) essential oils (EOs) (including cinnamon, clove, thyme, oregano, and orange terpene oils) at concentrations of 1–20% against the maize weevil using an olfactometer and a simulated fumigation method, respectively. The olfactory tests show that cinnamon oil had the highest repellency (90%) to the weevils among the EOs tested. The insecticidal activity study indicates that maize weevil mortality increased with EO concentration and storage time with cinnamon, clove, and thyme oils being more effective. No weevil death was observed at 1% EOs; weevil mortality was 3.3–36% at 5%, which varied with the type of EO and storage time. At 10% or higher concentrations, all tested EO showed comparable or higher insecticidal activity than pirimiphos methyl-positive control at its recommended concentration (5 mg/kg corn). No significant increase in weevil mortality was observed with further increase in EO concentration, with exceptions of oregano oil and thyme oil. The highest weevil mortality levels were observed at week 7 for 15% cinnamon oil (100%) and eugenol (100%), followed by 20% thyme oil (93%). The study indicates that some EOs have great potential to serve as synthetic insecticide alternatives to protect organic corn grains from maize weevil damage during storage. This is important to food security, safety and environmental health.

## 1. Introduction

Cereals are known for being the most important crops in the world and are indispensable for feeding the world. Important cereal grains include rice, corn, wheat, barley, oat, rye, sorghum, and millet, with rice, corn, and wheat being the three major cereal crops. The United States is the largest corn producer in the world, followed by China, Brazil, European Union and Argentina [1]. Organic food is a fast-growing, high-value market segment driven by consumer demand for healthy and nutritious food. The production of organic corn grains has tremendously increased in past two decades. Organic corn for human consumption or animal feed is grown using production methods intended to mimic natural processes that exclude most synthetic pesticides and standard commercial fertilizers [2].

In most places, crops are grown seasonally, but they are needed by end users throughout the year; thus, they must be stored for short or long periods after harvesting to meet the needs of the food supply chain and to save seeds for the next season. Insect infestation is one of the major challenges during cereal grain storage. Several studies have reported that maximum losses happen during this operation [3,4]. Losses as high as 59.48% have been estimated in maize grains after storing them for 90 days in traditional storage structures (granary/polypropylene bags) in sub-Saharan Africa [5]. The storage losses are affected by several factors, which can be classified into two main categories: biotic factors (insects, pests, rodents and fungi) and abiotic factors (temperature, humidity and rain). Among all the biotic factors, insects and pests are considered the most important and cause huge losses in grains (30–40%) [6]. Grain insects cause significant crop loss during post-harvest storage by direct feeding damage or deterioration and contamination of grains. The insects commonly known to infest stored grains are the granary weevil (*Sitophilus granaries*, L., Coleoptera: Curculionidae), the rice weevil (*Sitophilus oryzae* L., Coleoptera: Curculionidae), the maize weevil (*Sitophilus zeamais Motschulsky*, Coleoptera: Curculionidae), lesser grain borer (*Rhyzopertha dominica*, Coleoptera: Bostrichidae), and angoumois grain moth (*Sitotroga cerealella*, Olivier) [7]. The economic impact on farmers due to the loss of crops from insect infestation are an impairment of fertilization rates or seed recovery, and the application of pesticides can be harmful to the soil and water fertility [8]. The loss of grains during storage could lead to increases in grain prices, which can negatively affect consumers. The loss of storage grain due to insect infestation threatens the livelihood of small-scale farmers. Grains that are badly damaged have a strong odor mixed with debris and insects, which discourages potential buyers and leads to a loss in sale; damaged grains are used to feed animals, which is a huge economic loss to the household [9]. It has been estimated that 1% to 5% of stored grain in developed countries and 20% to 50% of stored grain in developing countries is lost due to insect damage [10]. This poses a negative impact on food security and nutrition in developing countries. In addition, insect infestation can contribute to heating in corn stored under tropical conditions. weevils are often associated with the production of large amounts of heat and metabolic water, which facilitates the growth of certain storage molds that greatly accelerate deterioration [11] and cause food safety issues because many storage molds produce mycotoxins that are harmful to both humans and animals.

Generally, an integrated approach is needed to control/manage insects during grain storage. For conventional cereal grains, the approach may include methods such as keeping bins clean and repaired, the fumigation of empty storage bins and surrounding areas with insecticide sprays, storing clean dry grain, applying a grain protectant (a labeled insecticide) on the grain as the grain stream is moved into the storage structure aeration, followed by a second insecticide application to the top of the leveled grain mass inside the bin or structure, inspecting the grain regularly [12,13]. Despite their usefulness, pesticides could pose potential risks to food safety, environment, and all living things [14]. Furthermore, the synthetic pesticides used for insect control in conventional cereal grain storage are not allowed in the case of organic grain storage. Therefore, there is a need to seek or develop safer alternatives for insect management during organic corn grain storage. Some studies have evaluated the insecticidal properties of EOs [15,16]. Many EOs have shown insecticidal properties, which means that the EO can kill or discourage the insects. The insecticidal properties of EOs varies with exposure time, insect species, and plant sources of EOs [15]. Considerable recent attention on EOs has focused on their broad range of bioactivities on pests and disease-causing organisms and thus their potential use as alternatives to synthetic pesticides for crop protection and in other pest management contexts [17]. However, most EOs have a strong odor, and it may result in an undesirable flavor if EOs are sprayed directly on the grain surface. Therefore, the purpose of this study is to evaluate the insect repellency and insecticidal activity of six different EOs including cinnamon oil, clove oil, eugenol, orange terpenes, oregano oil and thyme oil against maize weevil in stored organic corn grains using a simulated fumigation method by which the corn grains do not come into direct contact with EOs. The reason for selecting these EOs is that they are generally recognized as safe (GRAS) [18] and have been extensively studied for their antioxidant and antimicrobial activities in processed food products [19,20].

## 2. Materials and Methods

### 2.1. Materials

Organic corn grains were purchased from Great River Organic Milling (Arcadia, WI, USA). The maize weevil, *S. zeamais*, was kindly provided by Dr. Beatrice Dingha’s Lab at North Carolina A&T State University. Six essential oils (EOs) (including thyme oil, oregano oil, cinnamon oil, clove oil, eugenol, and orange terpenes) and dimethyl sulfoxide (DMSO) were purchased from Sigma-Aldrich (St. Louis, MO, USA). Pirimiphos-methyl, an insecticide specifically for fumigation of corn storage facility in the United States, was also purchased from Sigma-Aldrich and used as positive control, while 10% DMSO in deionized water was used to dilute EOs and served as negative control. Wide mouth clear glass jars (60 mL) with screw caps, Mason jars (1 L), glass serological pipets, disposable pipette tips, and cotton balls were purchased from Fisher Scientific (Suwanee, GA, USA).

### 2.2. Weevil Culture Preparation

The corn was inspected for the presence of weevils and mold before starting the weevil culture. Each Mason jar (1 L) was filled with 400 mL of corn grains. A mesh covering was created for the lid of each Mason jar to prevent the weevils from escaping and to ensure ventilation. Along with each mesh covering, filter paper was added to the lid to make sure it was properly closed. The adult weevils were taken from jars containing previously cultured weevils and added to the new jars with clean corn. To transfer the adult weevils from old corn to new corn, the mixture of old corn grains and weevils was poured from the old jar into a No. 10 sieve (we tried not to put a lot of the dust into the sift). The sieve was covered and shaken to remove corn dust caused by weevils. The weevils that remained on the screen of the sieve were put into the new jar with freshly added corn grains, covered with the mesh (size 20) covering and incubated in an environmental chamber for 2 weeks at room temperature and 60% relative humidity. A total of 6 jars of weevils were cultured once a week on three days a week (Mondays, Wednesdays and Fridays) to prevent the shortage of weevils and to ensure sufficient number of 1–3 days old weevils for the olfactometer, insecticidal, and synergistic experiments.

### 2.3. Preparation of EOs of Different Concentrations

Dimethyl sulfoxide (DMSO) was used to prepare the working solutions of EOs because DMSO is an important polar aprotic solvent that dissolves both polar and nonpolar compounds and is miscible in a wide range of organic solvents as well as water [21]. More importantly, DMSO is a non-volatile and non-toxic solvent with a median lethal dose higher than ethanol (DMSO: LD_50_ = 14,500 mg/kg, ethanol: LD_50_ = 7060 mg/kg in oral challenge with rat) (https://www.chemicalbook.com/ChemicalProductProperty_EN_CB7854105.htm accessed on 18 September 2020). DMSO is frequently used to dissolve test compounds in in vitro drug discovery and drug design and is used as a vehicle in the in vivo studies of test compounds because it could penetrate the skin and other membranes without damaging them and could carry other compounds into a biological system [22]. Briefly, 10 mL of DMSO was dissolved in 90 mL of deionized water to obtain 10% DMSO solution, which was used to dilute the EO. To prepare 1%, 5%, 10%, 15% and 20% essential oil working solutions, 0.1, 0.5, 1, 1.5 and 2 mL of essential oil were added to a set of 20mL amber glass vials containing 9.9, 9.5 and 9.0, 8.5 and 8 mL of 10% DMSO. The vial was capped immediately after adding EO, then mixed thoroughly using a vortex mixer to ensure complete mixing. The EO working solutions were prepared in a fume hood immediately before using.

### 2.4. Use of 4-Choice Arena Olfactometer to Evaluate Attractiveness or Repellency of Weevil

Various types of olfactory meters have been used for testing the repellent activity of essential oils against mosquitoes [23], rice weevils [24], moths [25] and horn flies [26]. Olfactory tests were conducted to evaluate the repellency or attractiveness of each EO to the maize weevils at different concentrations. A 6 × 4 two-factor factorial experimental design was used. The two factors were type of EOs (total 6) and concentration of EO (1, 5, 10 and 15% for each EO). The tests were conducted using a 4-Choice Arena Olfactometer with removable lid (Sigma Scientific, Micanopy, Florida). The olfactometer had a central chamber (30 cm long by 30 cm wide by 5 cm high) with four “arms” (7 cm long by 2 cm in diameter). The four openings (“arms”) of the olfactometer were connected by Teflon glass tube connectors to a clean air delivery system that pumped clean air into the four arms at a constant rate of 200 mL/min. One milliliter of essential oil (same concentration) was added onto cotton balls, which were placed into the two opposite arms of the olfactometer. The cotton ball without EO and DMSO was inserted into the third arm and used as blank, while the cotton ball with 10% DMSO was placed into the fourth arm and used as the negative control. Ten adult maize weevils were added at the “base” (the central bottom of chamber) of the olfactometer. The experiment was then started, and the movement of weevils was observed for an hour to find out which of the EOs attract or repel the weevils. The experiment was replicated five times using 1-3 day-old weevils.

### 2.5. Insecticidal Activity of Individual Essential Oil

Insecticidal activity tests were conducted to evaluate the effects of type and concentration of EO on the mortality of maize weevil. A two-factor factorial experimental design was used. The two factors were type of EOs (total 6 EOs) and concentrations of EO [five concentration (1, 5, 10, 15 and 20%) for each EO]. A published method [27] was modified for this study. Briefly, each essential oil was diluted to the desired concentrations with 10% DMSO in a set of amber glass vials. A set of glass jars with capacities of 60 mL were labeled with the names of each essential oil and their concentrations along with the date of the experiment. An amount of 20 g of corn was then added to the jar along with 10 adult maize weevils that were 1–3 days old. The diluted essential oil (1 mL) was added to the cotton ball taped on the lid of glass jar, the jar was capped immediately and stored at room temperature for 7 weeks. At the same EO concentration, the experiment was replicated 3 times. The number of dead insects was counted weekly, and the mortality of maize weevil was calculated. Weevil mortality was calculated using the number of dead weevils over the total number of weevils multiplied by 100. The 10% DMSO was used as negative control, and pirimiphos-methyl, an insecticide registered for use on stored corn and sorghum grains, was dissolved in water to 100 µg/mL and used as positive control at concentration of 5mg/kg corn grain [28].

### 2.6. Insecticidal Activity of Two Different EOs-Synergistic Effect

Based on the results from Section 3.5, two EOs (A and B) with high insecticidal activities but different chemical compositions were selected for the synergy tests. Two EOs were diluted to 10% with 10% DMSO, and then mixed at ratios of 25/75, 50/50 and 75/25 (*v/v*). The volumes of all mixtures were the same (5 mL); thus, the total EO concentration of each mixture was 10%. The insecticide activity of each mixture was tested as described in Section 2.5. Each of the two EOs at the same concentration (10%) were used as control.

### 2.7. Data Analysis

All data collected were expressed as mean ± standard deviation (n = 5 for olfactory tests, n = 3 for insecticidal activity tests). The attractive/repelling properties EOs to maize weevils and the effects of type and concentration of EO on maize weevil mortality were analyzed by two-way analysis of variance (ANOVA) and post hoc Tukey multiple comparison using SAS version 9.4 (SAS Institute, Cary, NC, USA).

## 3. Results

### 3.1. Effects of Type and Concentration of Essential Oil on Insect Repelling

**Figure 1:**
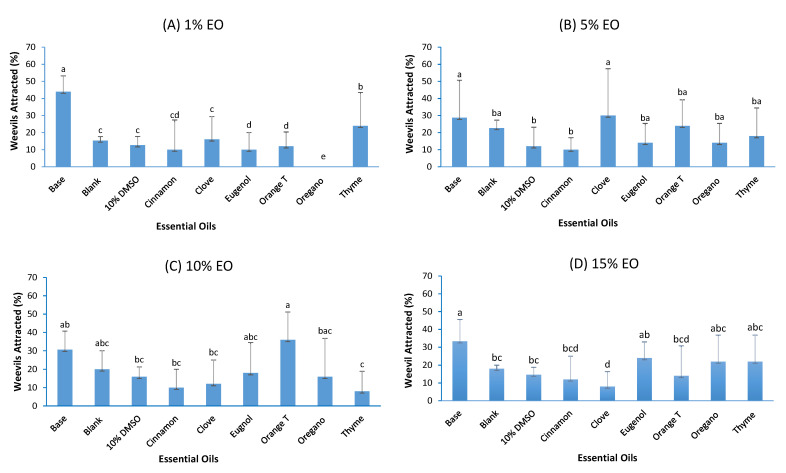
Percentages of maize weevils attracted to different essential oils at their different concentrations (Base—the bottom of the central chamber of olfactometer where the weevils were added. Blank is the negative control that did not have any EO or 10% DMSO on the cotton ball). Data bars with different labels are significantly different at 5% significance level.

The data presented in Figure 1 were expressed as percentage of maize weevil attracted by each EO at a specific concentration. The higher attracting power reflects lower repellency. Figure 1 shows that at the same EO concentration, the percentage of maize weevils attracted to different EOs varied greatly but not statistically significantly (F_5,23_ = 1.39, P = 0.2353), and the ability of each EO to attract/repel maize weevil varied with the EO concentration but did not increase with EO concentration (F_3,23_ = 1.03, P = 0.3810). At 1% EO, the majority of maize weevils remained at base (44%), thyme oil was most preferred by the maize weevils (24%) followed by clove oil, while oregano oil was least preferred (0%) (Figure 1A). At 5% EO concentration, the percentage of weevils that remained at the base decreased, those attracted to clove oil and orange terpenes increased, while those attracted to thyme oil decreased. Clove oil and orange terpenes were preferred by maize weevils, whereas cinnamon oil was the least preferred at a concentration of 5%. At higher EO concentrations (10% and 15%), the average percentage of weevils attracted by cinnamon oil and clove oil remained low (8–10%), while the percentage of weevils attracted by other EOs fluctuated greatly with EO concentrations. Therefore, cinnamon oil should have the highest maize weevil repelling capability among the EOs tested in this study.

### 3.2. Synergistic Effect of Cinnamon Oil and Clove Oil on Maize Weevil Repelling

**Figure 2:**
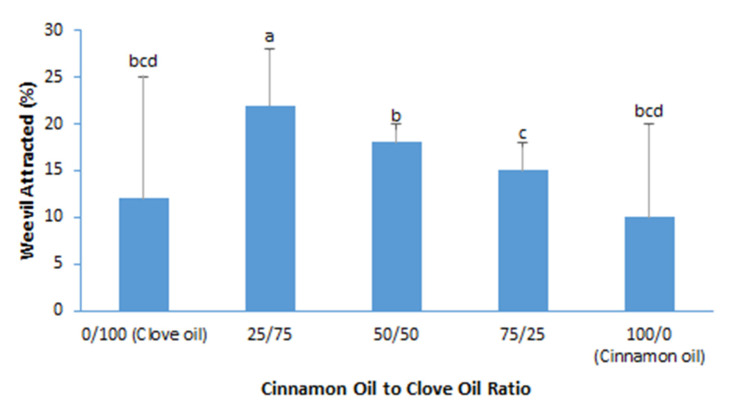
Percentage of maize weevil attracted to the mixtures of 10% cinnamon oil and clove oil. Data bars with different labels are significantly different at 5% significance level.

The purpose of conducting the synergy test was to find out if mixing two or more essential oils together could enhance the potential of insect repelling. If a mixture of two EOs exhibits higher insect repelling potential than a single EO at the same concentration, there is synergy between the two EOs. In this study, the synergy between cinnamon oil and clove oil was tested at ratios of 25/75, 50/50 and 75/25 at total a EO concentration of 10%, and 10% cinnamon oil and 10% clove oil were used as controls. Figure 2 shows that all mixtures of these two EOs attracted more weevils than cinnamon oil or clove oil. This suggests that these two EOs enhanced each other’s weevil attracting potential, but not weevil repelling potential.

### 3.3. Insecticidal Activities of Different Essential Oils

Table 1 is the result of analysis of variance of all maize weevil mortality data. The table shows that the mortality was significantly affected by the EO type, EO concentration and storage time (*p* < 0.0001). The weevil mortality was also significantly affected by the interaction between EO type and EO concentration (*p* < 0.0001), and the interaction between EO concentration and storage time (*p* < 0.0001). The interaction between EO type and storage time was not significant.

**Figure 3:**
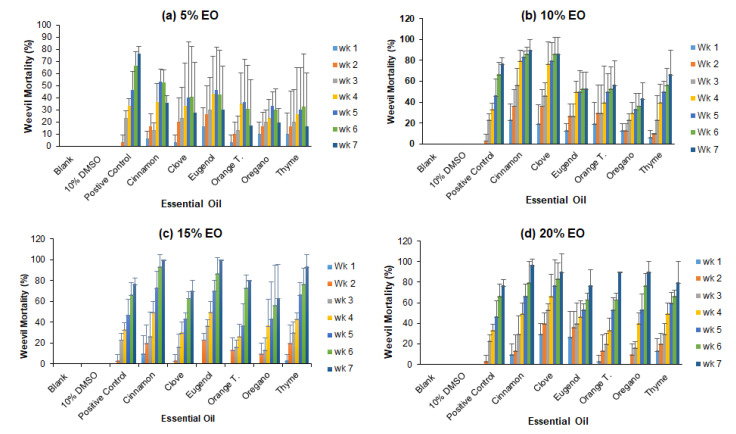
The mortality of maize weevils during storage in the presence of different essential oils.

Figure 3 depicts corn weevils’ mortalities caused by six essential oils at concentrations 5–20% over the course of seven weeks under simulated fumigation in glass jars containing organic corns. No weevil deaths were observed in the negative controls, indicating that DMSO was not toxic to maize weevils. Generally, as storage time increased, so did the mortality in the presence of EO or insecticide (positive control). At 5% EO, the mortality of the corn weevils observed weekly in the presence of cinnamon oil was higher than that caused by other EOs (*p* < 0.05), but lower than that caused by insecticide (*p* < 0.05). The highest corn weevil mortality was recorded in the presence of cinnamon oil at week 5 and week 6, with an average death of 53%. However, the mortality at week 7 seems lower than those at week 5 and 6, but they were actually not significantly different because of the large standard deviations of the experiments. At 10% EO, the highest mortality was observed in the presence of cinnamon oil and clove oil, while the lowest mortality was observed in the presence of oregano oil. On average, 90% and 87% were the highest mortalities observed at week 7 for cinnamon oil and clove oil, respectively. At an EO concentration of 15%, the number of dead weevils increased weekly over the 7-week storage period for all EOs. At the same storage time, treatments with cinnamon oil, eugenol and thyme oil had significantly higher corn weevil mortality than the positive control (*p* < 0.05), while treatments with clove oil, orange terpene, and oregano oil resulted in a weevil mortality similar to the positive control. On average, 100% of the corn weevils died at week 7 in the presence of cinnamon oil or eugenol. At 20% EO, cinnamon oil and clove oil resulted in higher weevil mortality than the positive control (*p* < 0.05), while other essential oils led to similar weevil mortality as positive control except for day 49, at which orange terpenes and oregano oil led to higher weevil mortalities than the positive control.

### 3.4. Effects of EO Concentration on Maize Weevil Mortality in Organic Corn Grains

**Figure 4:**
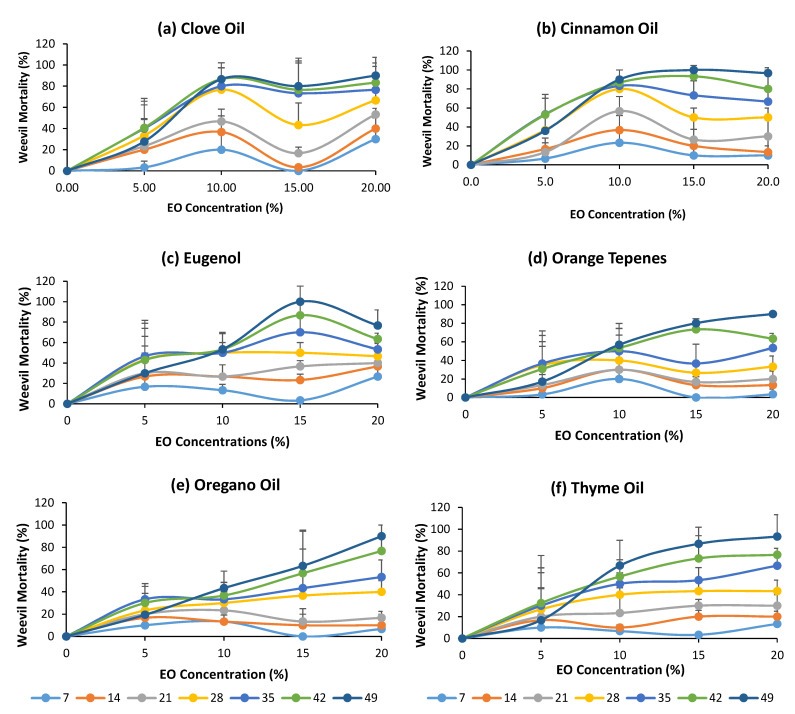
The mortalities of maize weevils in organic corn grains at different concentrations (%) of essential oils.

Overall, at lower concentration (5%), the insecticidal activity, expressed as maize weevil mortality, of tested EOs was lower than that of the positive control at the same storage time during a 7-week storage period, with exception of cinnamon oil, as shown in Figure 3a. The insecticidal activities of EOs increased with their concentrations (Figure 4). However, the highest maize weevil mortalities for cinnamon oil, clove oil and orange terpene treatments were achieved at 10% concentration, decreased at 15% and then increased slightly at 20% (Figure 4a,b). In other words, increasing their concentrations from 10% to 20% did not lead to a significant increase in the maize weevil mortality given the large standard deviations of the experiments. For other EOs tested, the maize weevil mortality increased steadily with increasing EO concentration and reached about same levels at 20% as that for cinnamon and clove oils at 10% (Figure 4c–f). Therefore, cinnamon oil and clove oil were more effective against maize weevil than other EOs tested in this study.

### 3.5. Insecticidal Synergy of Different Essential Oils

**Figure 5:**
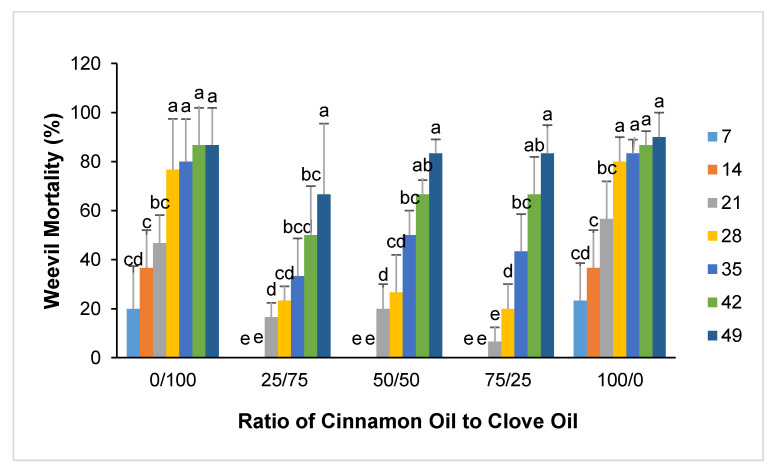
The mortality of maize weevils in organic corn grains at different cinnamon oil to clove oil ratios. Data bars with different labels are significantly different at 5% significance level.

Based on the results shown in Figure 3 and Figure 4, the maize weevil mortality caused by cinnamon oil and clove oil at a concentration of 10% was similarly high but below 100%. Thus, we selected cinnamon oil and clove oil to conduct synergy test at a total essential oil concentration of 10%. However, the results in Figure 5 did not show any positive synergy in insecticidal activity between cinnamon bark oil and clove oil. It can be seen from the figure that the mixtures of cinnamon oil and clove oil at different ratios resulted in lower mortalities of maize weevils than cinnamon oil or clove oil alone. Although the mortality increased with increasing cinnamon oil to clove oil ratio, it was not statistically significant.

## 4. Discussion

We experimented with the repellency and insecticidal activities of six essential oils, namely clove, eugenol, cinnamon, oregano, orange terpenes and thyme oils, on maize weevils. The repellent/attractive effects of these essential oils at different concentrations on stored maize weevils were measured using an olfactometer. Their corresponding insecticidal effects over the course of seven weeks were measured, with results for each week presented in this study. The synergistic effect of two combined essential oils were also experimented for its repellency/attractiveness and insecticidal effects on the weevils.

Generally, all the essential oils highly repelled the maize weevils compared to the controls, but the repellency differed among the essential oils tested in this study, with cinnamon oil being the most effective one. This might be due to the differences in the chemical composition of these essential oils. However, Patiño-Bayona and colleagues also found that chemical compositions of essential oils did not influence the repellent effect; thus, there was no differing effect among the different essential oils [16]. Additionally, the concentration of essential oils was inconsequential to the number of weevils repelled; that is, increasing the concentration of essentials oils did not produce a corresponding reduction in the attractiveness of essential oils to the weevils. Patiño-Bayona et al. (2021) experimented with the repellency of essential oils against *S. zeamais* and discovered great variations. They attributed the variations to the type of oil, exposure time, and oil concentration [16]. Lower doses of certain oils, for instance, 1% eugenol oil, produced a greater repellent effect on the weevils than at 5, 10, 15% concentrations. This conforms to the finding where lower doses of essential oils produced the greatest repellent effect [29]. Additionally, Mwangi and colleagues posit that increasing the concentration of *Capparis tomentosa* Lam. essential oil increased the repellent effect on maize weevils [30]. No synergistic effects of combined essential oils on insects’ repellency were observed in our study. In some cases, repellent effects on weevils were reduced when two essential oils were mixed. This antagonistic effect of combined essential oils, which resulted in less repellent effects than their pure compounds, was also observed by Noosidum and colleagues [31]. However, the study of Hategekimana and Erler found synergy or additive effects of binary and ternary combinations of EOs from anise (*Pimpinella anisum* L.), eucalyptus (*Eucalyptus camaldulensis* Dehn.) and peppermint (*Mentha piperita* L.) and their major components against rice weevil, but did not find antagonistic effects [22]

The insecticidal activity of all the six essential oils recorded every week over the course of a 7-week (49 days) storage period demonstrated palpable mortality against the weevils in the stored corn grains. As the maize storage time increased, a trend of increasing mortality of the weevils was observed, regardless of essential oil concentration. Several variations in insecticidal effects of the different essential oils over the course of 7 weeks were seen. The difference in insecticidal activities of different essential oils may be attributed to their different chemical compositions and insects’ susceptibility to those compounds [32,33]. Cinnamon oil produced the highest weevil mortality in the stored maize over the 7-week storage period at most concentrations tested in this study. In another study, cinnamon oil also demonstrated the greatest toxicity against rice weevils among other essential oils [34]. At 10%, about 90% of weevil mortality was observed from cinnamon oil treatment, and cinnamon oil resulted in the highest total mortality of weevils in the last week of storage at a concentration of 15%. The presence of trans-cinnamaldehyde in the cinnamon oil at the highest concentration, which is known to have an insecticidal effect on stored products, may be the reason for the high weevil mortality [15]. However, we also observed that most of EOs except thyme oil exhibited lower insecticidal activity at 15% concentration from day 7 to day 28 than that at 10% concentration. Similar results were reported in a recent study where EO extracted from *T. vulgaris CT geraniol* resulted in lower rice weevil mortality at 5% than at 4% [35].

The synergistic insecticidal effects between clove oil and cinnamon oil have also been studied. The chemical compositions of cinnamon bark oil and clove leaf oil are very different. The main constitute of EO extracted from *Cinnamomum zeylanicum* were found to be (E)-cinnamaldehyde (71.50%), linalool (7.00%), ß-caryophyllene (6.40%), eucalyptol (5.40%), and eugenol (4.60%) [36], while the major constitutes of EO extracted from clove oil leaf are eugenol (76.8%), followed by β-caryophyllene (17.4%), α-humulene (2.1%), and eugenyl acetate (1.2%) [37]. Thereby, we anticipated synergy between the main volatile compounds of cinnamon bark oil and clove oil used in this study. However, no synergistic effects were observed at different cinnamon oil to clove oil ratios. This finding was also observed in a study where the combination of tea tree oil with oregano oil, or the combination of thyme oil and oregano oil, did not have additive effects over those observed with tea tree oil alone [38]. The interactions obtained by combining agents may increase or decrease the magnitude of the effect depending on the individual agents combined [39]. No synergy of insecticidal activity of clove–cinnamon oil mixture may be because these two essential oils have the same mode of action, since synergy is a factor of multiple modes of action [40].

## 5. Conclusions

The GRAS essential oils (EOs) used in this study had both insect-repelling properties and insecticidal activity under the simulated fumigation conditions. Among all tested EOs, cinnamon oil showed constantly higher repellency to maize weevil, even at a very low concentration (1%). The cinnamon oil also showed higher toxicity to maize weevil than others at same concentration, followed by thyme oil and clove oil. Thus, they have the potential to serve as safe alternatives to synthetic pesticides to protect organic corn grains. However, further study is needed to scale up the approach in order to apply the EO in the grain storage setting on farm. In addition, the influences of EOs at their effective concentrations on the flavor of grains, particularly grains with higher lipid contents, needs to be investigated because most EOs have strong odors. Last but not least, the cost-effectiveness of using cinnamon oil, thyme oil or clove oil as insecticide alternative needs to be estimated according to the data obtained from the studies conducted in a larger storage setting similar to farm storage.

## Figures and Tables

**Table 1:** Analysis of variance (ANOVA) of maize weevil mortality as affected by the type of EO (EO-Type), concentration of EO (Conc) and storage time (Week).

Source	DF	Type III SS	Mean Square	F Value	Pr > F
**EO-Type**	6	399.628968	66.604828	21.34	<0.0001
**Conc**	3	199.307540	66.435847	21.29	<0.0001
**Week**	6	1869.822912	311.637152	99.86	<0.0001
EO-Type * Conc	15	222.228175	14.815212	4.75	<0.0001
EO-Type * Week	36	154.884921	4.302359	1.38	0.0775
Conc * Week	18	179.678571	9.982143	3.20	<0.0001
EO-Type * Conc * Week	90	122.702381	1.363360	0.44	1.0000

* in the table is used to express the interaction between two independent variables.

## Data Availability

Data is contained within the article.
